# Image Encryption Algorithm Based on Hyperchaotic Maps and Nucleotide Sequences Database

**DOI:** 10.1155/2017/4079793

**Published:** 2017-03-14

**Authors:** Ying Niu, Xuncai Zhang, Feng Han

**Affiliations:** College of Electric Information Engineering, Zhengzhou University of Light Industry, Zhengzhou 450002, China

## Abstract

Image encryption technology is one of the main means to ensure the safety of image information. Using the characteristics of chaos, such as randomness, regularity, ergodicity, and initial value sensitiveness, combined with the unique space conformation of DNA molecules and their unique information storage and processing ability, an efficient method for image encryption based on the chaos theory and a DNA sequence database is proposed. In this paper, digital image encryption employs a process of transforming the image pixel gray value by using chaotic sequence scrambling image pixel location and establishing superchaotic mapping, which maps quaternary sequences and DNA sequences, and by combining with the logic of the transformation between DNA sequences. The bases are replaced under the displaced rules by using DNA coding in a certain number of iterations that are based on the enhanced quaternary hyperchaotic sequence; the sequence is generated by Chen chaos. The cipher feedback mode and chaos iteration are employed in the encryption process to enhance the confusion and diffusion properties of the algorithm. Theoretical analysis and experimental results show that the proposed scheme not only demonstrates excellent encryption but also effectively resists chosen-plaintext attack, statistical attack, and differential attack.

## 1. Introduction

Using digital images to express information is intuitive, vivid, and informative; as a result, they have become a mainstream way of expressing information. With the widespread use of image information, ensuring security has become a universally concerning problem. Currently, digital image encryption technology has become an important method to protect the security of image information [1]. Due to a digital image's large data volume and high redundancy characteristics, the existing classical encryption methods cannot meet the needs of image encryption because of low efficiency of encryption and low security.

In 1949, Shannon put forward the concept of perfect secrecy and proved that the one-time pad cryptosystem had perfect secrecy, as discussed in his paper “Communication Theory of Secrecy Systems” [2]. However, the secret key of a one-time pad encountered a major difficulty in its transfer and distribution. According to pseudorandomness, sensitivity to an initial value, the predictive difficulty of a chaotic system, and the chaotic sequence can achieve the same encryption effect with a one-time pad as a random key, and in theory it is not broken. Chaotic encryption technology has been widely used in the field of information security, especially in the field of image encryption [3, 4]. Chen et al. offered a confusion and diffusion structure of an image encryption algorithm based on the chaotic system [4]. However, due to the limits of computer word lengths, the use of chaotic sequences can lead to chaotic dynamics degradation, especially for low-dimensional chaotic systems [5]. This can seriously impact the security of chaotic encryption. Therefore, to improve the security of the algorithm, many scholars have used a hyperchaos system to ensure the complexity of chaotic sequence. However, there is no denying that a single-encryption algorithm of chaotic mapping cannot guarantee the security of an encrypted image.

DNA is an important genetic information carrier in biology and plays an important role in the genetic organism metabolism. Its advantages include very large-scale parallelism, ultrahigh storage density, and low energy consumption; the unique molecular structure and molecular recognition mechanism of DNA determines its outstanding information storage and information processing ability. DNA molecules have great development potential in terms of information encryption, hidden certification, and other areas of information security technology [6–8], which provides a new way for developing modern cryptography [9]. In 1995, Dan et al. [9] cracked a 56-key code within 4 months, which demonstrated for the first time the understanding of traditional encryption standard (DES) with DNA computing. Afterwards, the development of DNA cryptography research became a hot topic of research. In 1999, Gehani et al. [10] used DNA as an information carrier and realized a one-time traditional encryption algorithm using biochemical technology in a DNA molecule. In the same year, Celland et al. [11] realized information hiding using DNA as an information carrier and hid the famous information “June 6 invasion: Normandy” in a DNA micropoint in World War II, thus realizing steganography based on the natural storage capacity of DNA. In 2013, Le Goff et al. implemented a three-dimensional (array particle) encryption model and successfully formed three-dimensional DNA hydrogel particle arrays within 100 microns in size by combining DNA particle technology with thermal shrinkage film to fix DNA polymers on a polyethylene heat-shrink chip [12]. The DNA encryption algorithm has been used to encrypt text messages, but it is very difficult to encrypt directly for image information. In 2015, Wu et al. found a new color image encryption scheme based on DNA sequences, and chaotic maps were proposed [13]. These algorithms only realized the location replacement of image pixels, which changed the gray value but failed to achieve the goal of true diffusion.

Therefore, this paper proposes a new image encryption algorithm by combining the chaotic system with the DNA code database. It replaces the bases under the displaced rules by using DNA coding in a certain number of iterations that are based on the enhanced quaternary hyperchaotic sequence, which is generated by Chen chaos; then, the algorithm conducts database operation with the DNA code base, thus further enhancing the confusion and diffusion properties of the algorithm through the cipher-text feedback and iterative chaotic systems.

## 2. Library Hyperchaos Sequence and DNA Sequence

### 2.1. Hyperchaos System and Hyperchaos Sequence Generation

**Figure 1:**
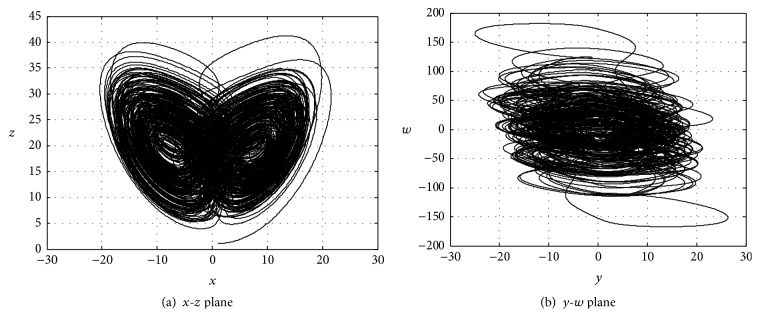
The attractor of the Chen chaotic system.

As a kind of special nonlinear phenomenon, chaos has a series of excellent features, such as good pseudo randomness, an unpredictable orbit, an extreme sensitivity to initial conditions, structural parameters, and nonrepetitive iterations; it has been widely used in secrecy communications. Compared with low-dimensional chaotic systems, high-dimensional chaotic systems have more positive Lyapunov exponents and are more complex, thus making it difficult to predict the dynamic characteristics that can effectively solve the degradation problem of low-dimensional chaotic system dynamics characteristics. High-dimensional chaos also provides strong confidentiality, a simple algorithm, and substantial key space. In 2005, Li et al. constructed a hyperchaos Chen system through the state feedback control:

(1) x˙=ay−x+ωy˙=dx−xz+cyz˙=xy−bzω˙=yz+rω,

where *x*, *y*, *z*, and *w* are system state variables and *a*, *b*, *c*, *d*, and *r* are system control parameters. The system performs hyperchaos movement when *a* = 35, *b* = 3, *c* = 12, *d* = 7, and 0.085 ≤ *r* ≤ 0.798. The attractor diagram of the system is shown in Figure 1 when *a* = 35, *b* = 3, *c* = 12, *d* = 7, *r* = 0.6, *x* = 1, *y* = 1.1, *z* = 1.2, and *w* = 1.3.

Four discrete real value hyperchaos sequences can be obtained through system iteration, wherein *A*_1_: {*a*_11_, *a*_12_,…, *a*_1*n*_}; *A*_2_: {*a*_21_, *a*_22_,…, *a*_2*n*_}; *A*_3_: {*a*_31_, *a*_32_,…, *a*_3*n*_}; and *A*_4_: {*a*_41_, *a*_42_,…, *a*_4*n*_}. For a uniform value range of the real number sequence, a new sequence can be obtained by taking a fractional part of the four sequences only, wherein *B*_1_: {*b*_11_, *b*_12_,…, *b*_1*n*_}; *B*_2_: {*b*_21_, *b*_22_,…, *b*_2*n*_}; *B*_3_: {*b*_31_, *b*_32_,…, *b*_3*n*_}; and *B*_4_: {*b*_41_, *b*_42_,…, *b*_4*n*_}.

(2) B1=A1−A1B2=A2−A2B3=A3−A3B4=A4−A4,

where [*x*] represents the integer part of *x*. One can calculate or replace the DNA sequence for convenience and define the quaternary hyperchaos sequence *P* as {*p*_1_, *p*_2_,…, *p*_*n*_}.

(3) $$\begin{array}{c} p_{i}=\left\{\begin{array}{cc} 0, & b_{1j}\leq b_{2j},\;\;b_{3j}\leq b_{4j};\\ 1, & b_{1j}\leq b_{2j},\;\;b_{3j}>b_{4j};\\ 2, & b_{1j}>b_{2j},\;\;b_{3j}\leq b_{4j};\\ 3, & b_{1j}>b_{2j},\;\;b_{3j}>b_{4j}. \end{array}\right. \end{array}$$

The quaternary hyperchaos sequence generated by this method can eliminate the correlation between adjacent elements in the chaotic sequence and demonstrate good random distribution.

### 2.2. Nucleotide Sequences

A DNA molecule is composed of four DNA nucleotides, which are adenine (A), cytosine (C), guanine (G), and thymine (T). Two single DNA molecules can form a stable DNA molecule through hydrogen bonds between the nucleotides. The chemical base structure determines the principle of complementary base pairing, which is known as the Watson-Crick base pairing principle, namely, A bonds with T using two hydrogen atoms and G bonds with C using three hydrogen atoms [14, 15]. The natural combination of a quaternary is similar to binary semiconductor switching. Therefore, information storage and computing can be completed using base permutation and combination [16].

The nucleic acid database is an information collection database that contains the nucleotide sequence of nucleic acids and their polymorphisms, structures, properties, and other related descriptions about a single nucleotide. The database file can be accessed from the biological information resource center through any computer network. A sequence ID in the database is called a sequence code; it is unique and permanent.

With the rapid development of sequencing technology, the scale of the nucleic acid database is growing; the index doubled in size in less than nine months. In January 1998, there were 15500 sequence species included in EMBL, and the sequence number is currently more than one million, more than 50% of which are biological sequences. The number of open DNA sequences is more than 163 million to date [17].

Such a large-scale database is equivalent to a natural password, which provides a new train of thought and possible solutions for image encryption technology.

In the image encryption algorithm, three base algorithms are defined to achieve the purpose of pixel confusion and diffusion.

*(1) Encoding Rule*. If the corresponding code is carried on as A → 00, C → 01, G → 10, and T → 11, the complementary digital match 00↔11 and 01↔10 fits the complementary base pairs matching A↔T and C↔G. A total of 8 encoding combinations meet the complementary pairing rule [18, 19].

For a gray image, each pixel gray value can be expressed with an 8-bit binary number. If DNA code is used, only 4 base sequences are needed. The DNA sequence transformation rules can be used in image processing when they are converted into DNA sequences. To reach the goal of pixel value disturbance, the following base operation and transformation rules are defined at the same time in the encrypted image.

*(2) Base Algorithm*. According to the complementary pairing rules, there is an algorithm between bases (see Tables 1–3) for A → 00, C → 01, G → 10, and T → 11. Similar algorithms can also be built for other coding.

*(3) Base Substitution Rule*. For base transformation, we introduce a mapping function *L*(*x*) and make the agreement as follows:

(4) x≠Lx≠LLx≠LLLxx=LLLLx,

where *x*{A, C, G, T}. According to this agreement, there are 6 kinds of reasonable base substitution combinations (see Table 4).

In the pixel value replacement, we can select a replacement combination randomly and perform base displacement to achieve the goal of pixel value disturbance.

## 3. Encryption Algorithms

In this paper, the digital image encryption is realized by using two kinds of chaotic sequences, the DNA sequence library, and its pixel gray value transformation and operation, to achieve the purpose of confusion and diffusion.

### 3.1. Lorenz Chaotic Mapping

Lorenz mapping is a typical chaotic mapping in chaotic systems, and the system dynamic equation is

(5) x˙=αy−xy˙=−xz+βx−yz˙=xy−γz.

Among them are system parameters, and their typical values are separately 10, 28, and 8/3. When they are invariable, the system goes into chaos under the condition of 24.74.

The chaotic sequence system structure generated by the Lorenz system is more complex than the low-dimensional one, which can produce a combination of univariate or multivariate chaotic sequences. The sequence design is very flexible. The system can generate three chaotic real value sequences *x*, *y*, and *z* when *A*, the initial value, is given. Arranging them in ascending order obtains three new sequences *x*′, *y*′, and *z*′. Determining the location of each element for the chaotic sequence *x*, *y*, and *z* in the new ordered arrangement *x*′, *y*′, and *z*′ forms a replacement address collection index sequence *X*, *Y*, and *Z*. The index sequences are mainly used for scrambling the image pixel position matrix. Three indexes can realize three-pixel matrix scrambling.

### 3.2. DNA Sequences

**Figure 2:**
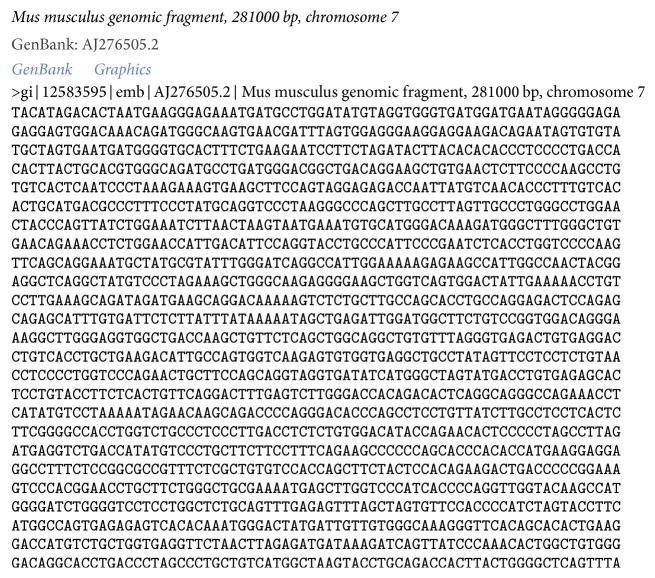
DNA sequence ID in GenBank for AJ276502.

Gray value diffusion is an essential step in the process of image encryption. In this paper, the pixel gray values are changed through the database operation between the image pixel gray value and the nucleic acid bases of the DNA sequences. As an example, this paper adopts the DNA sequence ID in GenBank for AJ276502, which contains 281000 bp bases, as shown in Figure 2. The base sequence information downloaded from the website is shown in the figure. In the image pixel gray value computation, the starting base location *R* can be determined randomly, such as *R* = 101.

### 3.3. Encryption Algorithm Design

**Figure 3:**
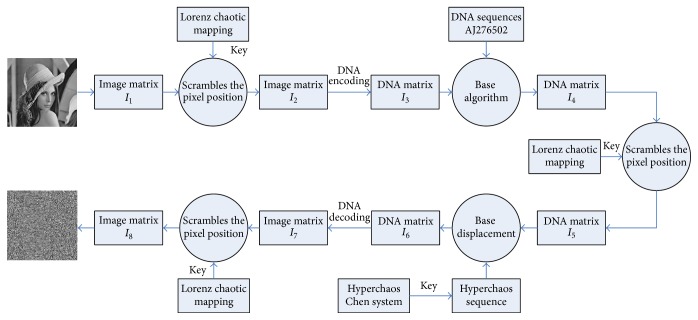
Description of the encryption process.

In this paper, the digital image encryption algorithm is divided into two parts. The first part is the pixel position scrambling transformation. The image pixel location will be changed through the displacement index created by the Lorenz chaotic sequence. The second part is the pixel gray value transform and spread. We transform the value of each original image pixel into DNA sequences, operate with the sequence in the DNA coding sequence database, and then perform iteration replacement through the cipher-text feedback. The encryption process flow diagram is shown in Figure 3. Specific steps are as follows:

Input: gray image *I*, parameters of the initial value.
Output: encrypted image.
(1) Convert the gray-scale image *I* to two-dimensional *M* × *N* matrix *I*_1_.
(2) Scramble the image pixel position matrix *I*_1_ according to the index sequence *X* produced by Lorenz map and obtain the image pixel location matrix *I*_2_.
(3) Encode each pixel to DNA four-base sequence under a random DNA encoding rule to obtain a new DNA encoding matrix *I*_3_.
(4) Download the DNA sequence whose ID number is AJ276502 from GenBank database. Intercept *M* × *N* × 4 base sequences from *R* and translate them into matrix *I*′.
(5) Perform exclusive operation between *I*_3_ and its corresponding base sequence in *I*′, and then conduct addition operation with the previous pixel cryptograph to obtain new matrix *I*_4_. To obtain the coding matrix *I*_5_, scramble the code matrix *I*_4_ using the index sequence *Y* produced by three-dimensional Lorenz chaos system.
(6) Produce the *M* × *N* × 4 base DNA sequence *P* using hyperchaos Chen system, and choose the number of the corresponding base displacement in *I*_5_ according to the value of *p*_*i*_ and formula (6). Select a random rule from Table 4 to conduct base displacement and obtain the coding matrix *I*_6_.
(7) After replacement, choose a DNA encoding rule to convert bases to binary code, and further convert them into a decimal gray value to be *M* × *N* matrix and obtain the matrix *I*_7_. The displacement method is as follows:
  (6) xi=xi;pi=0xi=Lxi;pi=1xi=LLxi;pi=2xi=LLLxi;pi=3.
(8) Scramble the image pixel location matrix *I*_7_ according to the index sequence *Z* produced by Lorenz map and obtain the encryption image matrix to export.

The decryption process is an inverse algorithm. Therefore, we will not illustrate it in this paper.

This algorithm can also be applied to color image encryption; merely conducting RGB decomposition on the values of pixels is sufficient.

## 4. Experimental Result

In view of the algorithm proposed in this paper, the feasibility of the algorithm is verified in MATLAB software. This paper adopts the 256*∗*256 Lena gray images.

**Figure 4:**
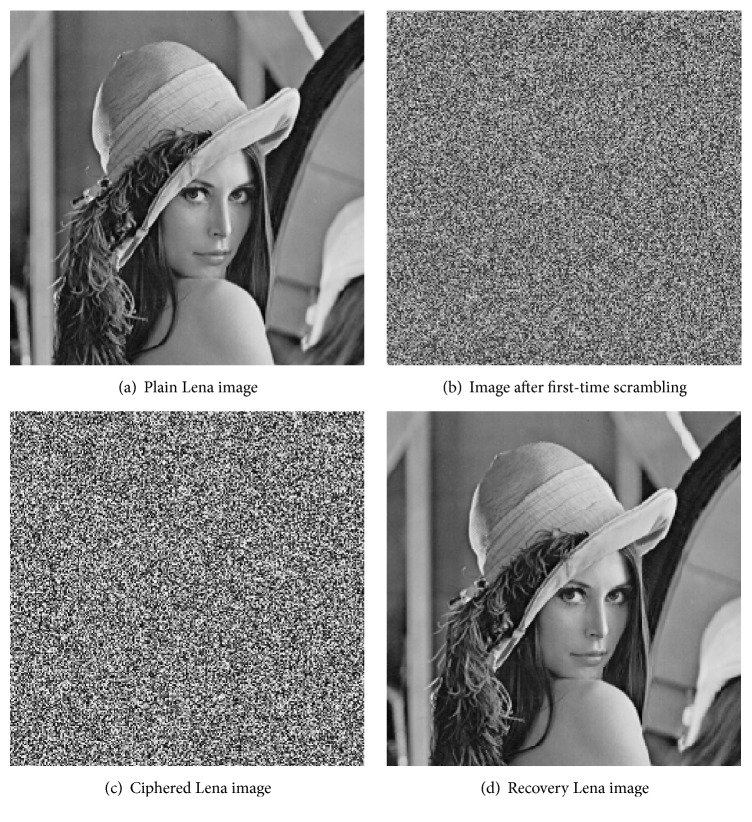
Lena image and ciphered Lena.

The original image and encryption image are shown in Figure 4.  Figure 4(b) is the image after first-time scrambling, and we cannot identify any of the original image information from it.

## 5. Security Analyses

### 5.1. Key Space and the Sensitivity Analyses

The keys in this paper are mainly used for pixel scrambling and the diffusion process: the preliminary Chen system is *x*_0_ = *y*_0_ = *z*_0_ = *w*_0_ = 1*e* − 6 and *r* = 0.6; the preliminary Lorenz chaotic mapping is *x*_0_′ = 0.0006, *y*_0_′ = −0.0006, and *z*_0_′ = −0.0006; rule 2 is selected as DNA encoding rule; rule 1 is selected as substitution rule; and the DNA sequence ID number is AJ276502 and the starting position is *R* = 1.

If the calculation accuracy is 10^−14^, the key space will reach 10^100^, which shows that this algorithm has sufficient space to resist a brute force attack.

**Figure 5:**
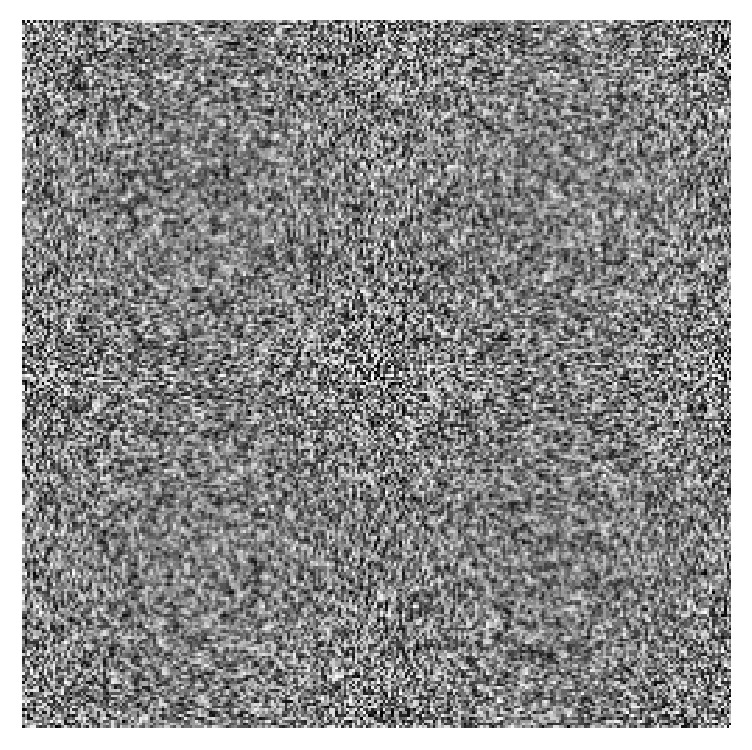
Failure of decryption of Lena.

To test the key sensitivity, we increase the initial value of *x*_0_′ for the Lorenz map to 0.0000000001 and keep the other keys invariant.  Figure 5 shows the corresponding decryption figure. It shows that the key cannot decrypt the original image correctly when the key changes slightly, which indicates the algorithm has stronger key sensitivity.

### 5.2. Gray Histogram Analysis

**Figure 6:**
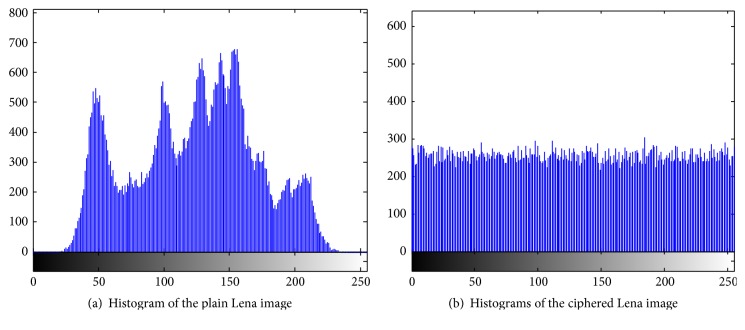
Histogram of the plain Lena image and ciphered Lena image.

The original image gray value distribution can be exposed to a certain extent from image statistics; therefore, it is vitally important to change the statistical distribution of the original image. The image pixel gray value arithmetic operation is used for the purpose of a gray statistical attack defense. As shown in Figure 6, experiment results show that exclusive processing and replacement operation make the encrypted image gray-scale distribution uniform, which demonstrates that the algorithm has a very good ability to resist statistical analysis; attackers will not be able to analyze the original gray value distribution.

### 5.3. Correlation Analysis

**Figure 7:**
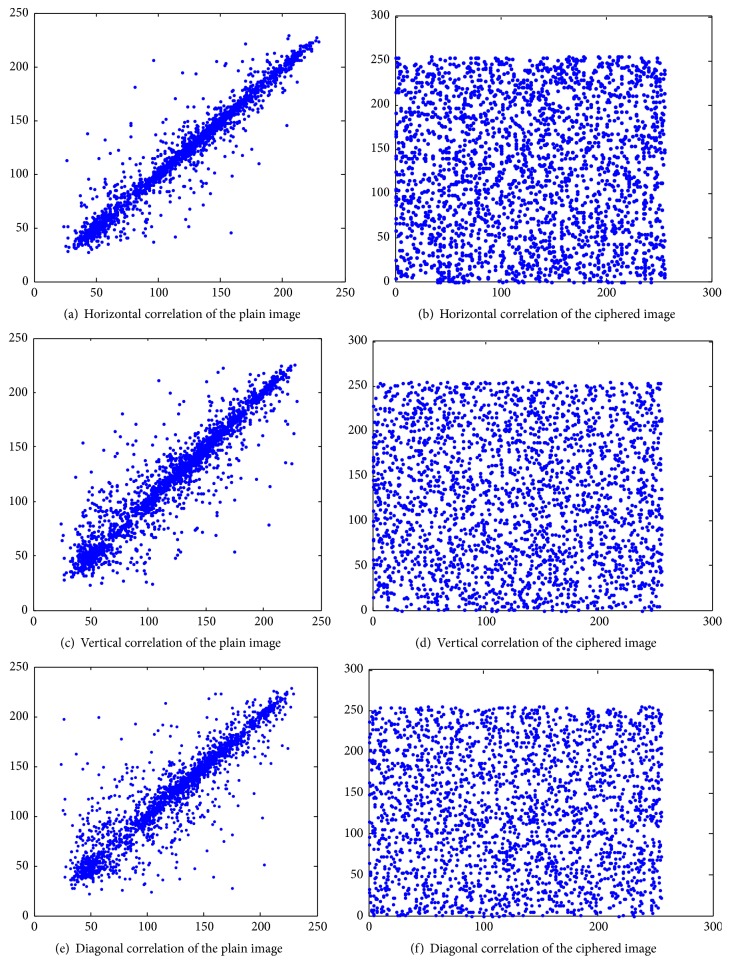
Correlation analysis of Lena as a ciphered image in three directions.

The original image pixel correlation is usually large, so we must prevent the reduction of the correlation of adjacent pixels to prevent statistical analysis. An encrypted image and its original image are selected randomly for 2500 pixels, and the horizontal, vertical, and diagonal pixel correlation results are shown in Table 5. Table 5 shows that former image pixels have great correlation, which greatly reduces after encryption compared with before. This suggests that the adjacent pixels have been largely irrelevant, and the statistical characteristics of the original image have been spread randomly to cipher-text images. Table 5 and Figure 7 present correlation comparisons of adjacent pixels between the original and encrypted images.

### 5.4. Information Entropy Analysis

The information entropy is a type of index for an uncertainty test. Its computation formula is as follows:

(7) $$\begin{array}{c} H\left(\;m\;\right)=-\overset{2^{N}-1}{\underset{k=0}{\sum }}p\left(\;m_{i}\;\right)\log_{2}p\left(\;m_{i}\;\right), \end{array}$$

where *p*(*m*_*i*_) is the appearance probability of information. For gray images, there are 256 information states for information *m*. The minimum value of *m* is 0, and the maximum is 255. According to the former formula, the information is completely random when the information entropy is 8. That is, the bigger the cipher-text information entropy is, the more security the information has. In this paper, the information entropy for the Lena image is 7.9888, which indicates that the cipher-text information leakage is minimal. This further proves the security of this algorithm.

## 6. Conclusions

This paper presents a type of digital image encryption technology based on hyperchaos mapping and DNA sequence library arithmetic to realize a scrambling position transformation of image pixels and the spread of the pixel values. A safety analysis shows that the algorithm can effectively resist plaintext attack, differential attack, and statistical attack. Additionally, it provides a large key space and high security.

## Figures and Tables

**Table 1:** The XOR operation for DNA sequences.

XOR	A	C	G	T
A	A	C	G	T
C	C	A	T	G
G	G	T	A	C
T	T	G	C	A

**Table 2:** The addition operation for DNA sequences.

ADD	A	C	G	T
A	A	C	G	T
C	C	G	T	A
G	G	T	A	C
T	T	A	C	G

**Table 3:** The subtraction operation for DNA sequences.

Sub	A	C	G	T
A	A	T	G	C
C	C	A	T	G
G	G	C	A	T
T	T	G	C	A

**Table 4:** Base substitution rule.

1	$A\overset{L}{\to }T\overset{L}{\to }C\overset{L}{\to }G\overset{L}{\to }A$
2	$A\overset{L}{\to }T\overset{L}{\to }G\overset{L}{\to }C\overset{L}{\to }A$
3	$A\overset{L}{\to }C\overset{L}{\to }T\overset{L}{\to }G\overset{L}{\to }A$
4	$A\overset{L}{\to }C\overset{L}{\to }G\overset{L}{\to }T\overset{L}{\to }A$
5	$A\overset{L}{\to }G\overset{L}{\to }T\overset{L}{\to }C\overset{L}{\to }A$
6	$A\overset{L}{\to }G\overset{L}{\to }C\overset{L}{\to }T\overset{L}{\to }A$

**Table 5:** Adjacent pixels correlation comparison.

	Original image	Encryption image
Horizontal direction	0.9700	−0.0207
Vertical direction	0.9384	−0.0176
Diagonal direction	0.9176	0.0168
